# *Escherichia coli* triggers α-synuclein pathology in the *LRRK2* transgenic mouse model of PD

**DOI:** 10.1080/19490976.2023.2276296

**Published:** 2023-11-27

**Authors:** Dongxiao Liang, Han Liu, Ruoqi Jin, Renyi Feng, Jiuqi Wang, Chi Qin, Rui Zhang, Yongkang Chen, Jingwen Zhang, Junfang Teng, Beisha Tang, Xuebing Ding, Xuejing Wang

**Affiliations:** a Department of Neurology, the First Affiliated Hospital of Zhengzhou University, Zhengzhou, Henan, China; b Henan Key Laboratory of Chronic Disease Prevention and Therapy & Intelligent Health Management, Zhengzhou, Henan, China; c Department of Neurology, Multi-Omics Research Center for Brain Disorders, the First Affiliated Hospital, University of South China, Hengyang, Hunan, China; d Department of Neurology, National Clinical Research Center for Geriatric Disorders, Xiangya Hospital, Central South University, Changsha, Hunan, China

**Keywords:** Parkinson’s disease, *LRRK2*, *Escherichia coli*, gene-environment interplay, α-synuclein pathology

## Abstract

Alpha-synuclein (α-syn) pathology is the hallmark of Parkinson‘s disease (PD). The leucine-rich repeat kinase 2 (*LRRK2*) gene is a major-effect risk gene for sporadic PD (sPD). However, what environmental factors may trigger the formation of α-syn pathology in carriers of *LRRK2* risk variants are still unknown. Here, we report that a markedly increased abundance of *Escherichia coli* (*E*. *coli*) in the intestinal microbiota was detected in *LRRK2* risk variant(R1628P or G2385R) carriers with sPD compared with carriers without sPD. Animal experiments showed that *E*. *coli* administration triggered pathological α-syn accumulation in the colon and spread to the brain via the gut-brain axis in *Lrrk2* R1628P mice, due to the co-occurrence of *Lrrk2* variant-induced inhibition of α-syn autophagic degradation and increased phosphorylation of α-syn caused by curli in *E*. *coli*-derived extracellular vesicles. Fecal microbiota transplantation (FMT) effectively ameliorated motor deficits and α-syn pathology in *Lrrk2* R1628P mice. Our findings elaborate on the mechanism that *E. coli* triggers α-syn pathology in *Lrrk2* R1628P mice, and highlight a novel gene-environment interaction pattern in *LRRK2* risk variants. Even more importantly, the findings reveal the interplay between the specific risk gene and the matched environmental factors triggers the initiation of α-syn pathology in sPD.

## Introduction

Parkinson’s disease (PD) is the second most common progressive neurodegenerative disease and neuropathologically characterized by the accumulation of intraneuronal alpha-synuclein (α-syn) known as Lewy bodies.^1^ It is well established that pathological α-syn formation plays a central role in the pathogenesis of PD.^2^ Despite tremendous progress in this field, the exact pathways and mechanisms responsible for the initiation and progression of pathological α-syn formation remain unclear.^3^ Therefore, elucidating the molecular mechanisms underlying the pathological α-syn formation is particularly crucial for the diagnosis, treatment, and prevention of PD. Pathological α-syn aggregation in diverse regions corresponds to the development of both motor and non-motor symptoms of PD.^2,4^ A growing body of research has begun to reveal that the onset of PD is believed to be localized to peripheral organs, particularly the gastrointestinal (GI) tract.^5–7^ Postmortem histopathological studies have revealed the widespread distribution of pathological α-syn in the GI tract of patients with PD, which potentially corresponding to the major non-motor symptoms, constipation. It occurs long before the appearance of motor symptoms.^8^ The spreading of pathological α-syn from the gut to the brain through the vagus nerve, inducing PD-like symptoms, was confirmed in mouse models of PD.^7^ These studies suggest that α-syn pathology may originate in the gut and spread to the brain along the gut-brain axis. However, it is still unknown why and how pathological α-syn aggregates is generated in the gut.

The leucine-rich repeat kinase 2 (*LRRK2*) gene is one of the best-known genetic contributors to sporadic PD (sPD).^9,10^ *LRRK2* R1628P and G2385R are the most common susceptibility variants that increase the risk of sPD in Asiatic populations.^11,12^ However, 2% and 4.7% of healthy individuals carry the R1628P and G2385R risk variants of *LRRK2*, respectively.^13,14^ Thus, the effects of both genetic and environmental factors must co-trigger the pathogenesis of sPD in carriers of *LRRK2* risk variants. The matched environmental factors of *LRRK2* risk variants and their specific interplay underlying sPD remain unknown. Therefore, the following question arises: whether *LRRK2* risk variants, acting as genetic factors, interact with their corresponding environmental factors to contribute substantially to the occurrence of *LRRK2*-associated sPD? Interestingly, genome-wide association studies have identified that *LRRK2* gene is also a common susceptibility locus for Crohn’s disease (CD), a chronic inflammatory disease of the GI tract.^15,16^ Moreover, several epidemiological studies have shown that patients with CD had an increased risk of PD, and had higher levels of LRRK2 in inflamed colonic tissue.^17,18^ In addition, individuals carrying *LRRK2* risk variants were highly susceptible to GI disturbances, mainly constipation.^19^

The microbiota-gut-brain axis bidirectionally transmits information between gut and brain.^20^ A variety of studies in preclinical animal models have offered support for the notion that disturbances in the composition of the intestinal microbiota may play an important role in brain disorders.^21,22^ Recently, increasing evidence has demonstrated the intestinal microbiota abnormalities in patients with sPD and mouse models of PD.^22,23^ Changes in the intestinal microbiota can be one of the key initiating events in PD pathogenesis.^24^ Together, all of these led us to hypothesize that the onset of *LRRK2*-associated sPD is dependent on both an external factor (intestinal microbiota) and a genetic component (*LRRK2* risk variants). However, much less is known about whether intestinal microbiota dysbiosis is involved in *LRRK2*-associated sPD.

In the present study, we found that a significantly increased relative abundance of *Escherichia coli* (*E. coli*) in the intestinal microbiota was detected in *LRRK2* risk variant (R1628P or G2385R) carriers with sPD (*LRRK2+*/sPD+) compared with the *LRRK2* risk variant (R1628P or G2385R) carriers without sPD (*LRRK2+*/sPD-). Animal studies demonstrated that the administration of *E. coli* to *Lrrk2* R1628P mice initiated the formation of α-syn pathology in the colon, and then α-syn aggregates were transmitted to the brain through the gut-brain axis, inducing PD-like symptoms. Furthermore, we found *E*. *coli*-derived extracellular vehicles (EVs) act carriers to deliver curli into the colonic mucosal epithelial cells, and greatly enhanced DAPK1 expression to enhance the Ser129-phosphorylated α-syn. Meanwhile, *LRRK2* R1628P inhibited autophagic degradation of α-syn. Amazingly, fecal microbiota transplantation (FMT) showed great potential for improving movement symptoms and mitigating α-syn pathology in *Lrrk2* R1628P mice. Overall, we demonstrate that *E. coli* triggers α-syn pathology in *Lrrk2* R1628P mice and these findings highlight a novel gene-environment interaction pattern between *LRRK2* risk variants and *E. coli* in the pathogenesis of *LRRK2*-associated sPD.

## Results

### Intestinal microbiota dysbiosis in LRRK2 risk variant carriers with sPD

**Figure 1.**
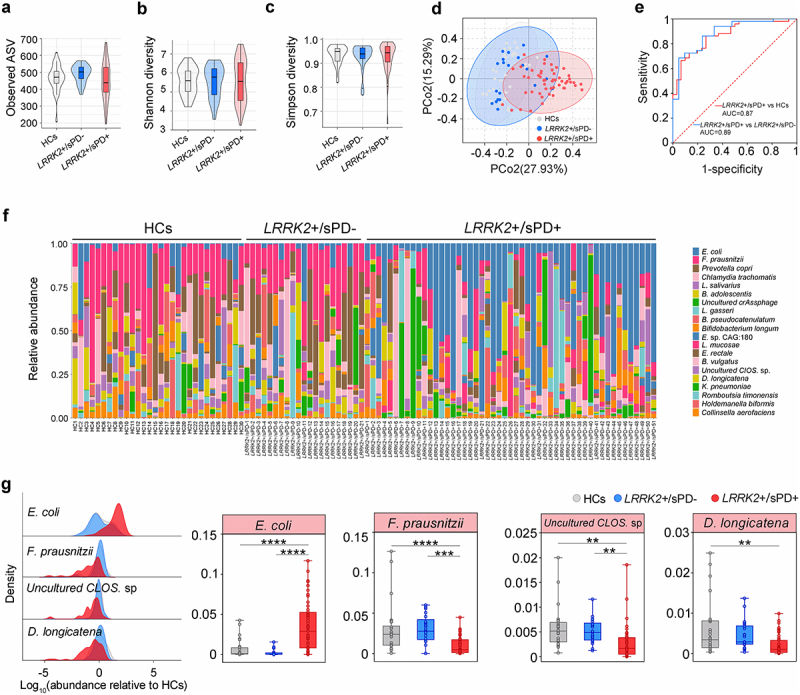
Dysbiosis of intestinal microbiota in *LRRK2*-associated sPD. (a-c) Boxplots of α-diversity as measured by observed number of ASVs (a), Shannon diversity (b), and Simpson diversity (c) from the fecal microbiota at genus level in terms of 16S rDNA amplicon sequencing among *LRRK2*+/sPD+, *LRRK2*+/sPD- and HCs. Box elements show the median, minimum, and maximum values. (d) PCoA plot based on Bray-Curtis dissimilarities of the fecal microbiota at genus levels in terms of 16S rDNA sequencing among *LRRK2*+/sPD+, *LRRK2*+/sPD-, and HCs. Ellipses show 95% confidence intervals. (e) ROC analysis based on the levels of *E*. *coli* for two-way classification models of *LRRK2*+/sPD+, *LRRK2*+/sPD- and HCs. (f) Bar plots of the species levels in fecal samples based on metagenomic sequence analysis among *LRRK2*+/sPD+ (*n* = 51), *LRRK2*+/sPD- (*n* = 21), and HCs (*n* = 30). (g) Relative abundance distributions of fecal microbiota at species levels in terms of metagenome sequencing among *LRRK2*+/sPD+, *LRRK2*+/sPD-, and HCs (left). Data are expressed as the median, minimum, and maximum (right). The following *n* values represent the number of independent individuals for statistical evaluation: (a-e, g), *LRRK2*+/sPD+ = 51, *LRRK2*+/sPD- = 21, HCs = 30. Data are analyzed by Permutational Multivariate analysis of Variance (PERMANOVA) (d), one-way ANOVA with Tukey’s multiple comparison test (a-c, e), and covariate-adjusted random coefficient regressions (g). *p < .05, **p < .01, ***p < .001, ****p < .0001.

To map the composition of the intestinal microbiota in *LRRK2+*/sPD+ (R1628P, *n* = 20; G2385R, *n* = 31), *LRRK2+*/sPD- (*n* = 21) and healthy controls (HCs, *n* = 30), we first used high-throughput sequencing to amplify the V3-V4 regions of the 16S ribosomal DNA (16S rDNA)^25^ from human fecal samples. The results of principal coordinate analysis (PCoA) at the genus level in fecal microbiota composition showed that the samples from all the participants were mainly scattered into three clusters, with the highest diversity in *LRRK2+*/sPD+ (Figure 1d). However, neither the Shannon indices, the Simpson diversity nor the observed amplicon sequence variants (ASVs) were significantly different among *LRRK2+*/sPD+, *LRRK2+*/sPD- and HCs (Figure 1a–1), respectively. Subsequently, shotgun metagenome sequencing^26^ was used to analyze more accurate sequence-based information of species in human fecal samples (*LRRK2+*/sPD+, *n* = 51; *LRRK2+*/sPD-, *n* = 21; HCs, *n* = 30). The data showed that the fecal microbial species were altered in both distribution and abundance in samples from *LRRK2+/*sPD+ compared with *LRRK2+*/sPD- and HCs (Figure 1f), respectively. We identified the top 20 species based on the mean relative abundance across all fecal samples (Figure 1f, Supplementary Table S1). Among the top 20 species, the mean relative abundances of 5 species in *LRRK2*+/sPD+ were significantly different from those in *LRRK2*+/sPD- and HCs, using covariate-adjusted random coefficient regressions with the adjustment of covariates (Supplementary Table S1). In addition, 4 species met a strict statistical threshold for significance (*P* < .01) in *LRRK2*+/sPD+ compared to *LRRK2*+/sPD- and HCs, including increased *E*. *coli*, and decreased *Faecalibacterium prausnitzii* (*F*. *prausnitzii*), *Uncultured Clostridium* sp. (*Uncultured CLOS*. sp.), and *Dorea longicatena* (*D. longicatena*) (Figure 1g, Supplementary Table S1). Notably, the relative abundance of *E*. *coli* in *LRRK2+*/sPD+ was the highest among the three groups. Area under receiver operating characteristic (ROC) curves^27^ were used to assess the sensitivity and specificity for distinguishing *LRRK2*+/sPD+ from *LRRK2*+/sPD- and HCs. The ROC curve analysis showed that the mean relative abundance of *E*. *coli* enabled us to distinguish *LRRK2*+/sPD+ from *LRRK2*+/sPD- and HCs with adequate accuracy (Figure 1e).

### Intestinal dysfunctions in Lrrk2 R1628P mice

**Figure 2.**
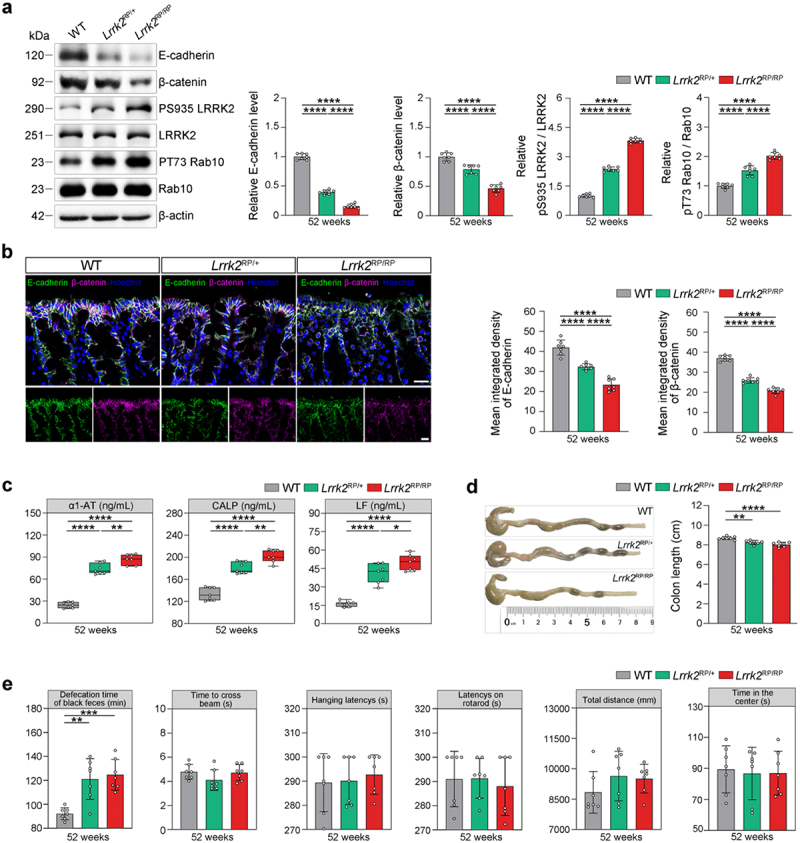
*Lrrk2* R1628P transgenic mice exhibit intestinal dysfunctions due to the pathological activation of LRRK2. (a) Representative immunoblot images of E-cadherin, β-catenin, pS935 LRRK2, total LRRK2, pT73 Rab10, and Rab10 levels in the colons of WT, *Lrrk2*^RP/+^, and *Lrrk2*^RP/RP^ at 52 weeks (left) and quantification (right). (b) Representative immunofluorescence images of E-cadherin (green) and β-catenin (magenta) in the colons of WT, *Lrrk2*^RP/+^, and *Lrrk2*^RP/RP^ at 52 weeks (left) and quantification of E-cadherin and β-catenin fluorescence intensity. (c) ELISA quantification of α1-AT, LF and CALP in fecal samples of WT, *Lrrk2*^RP/+^, and *Lrrk2*^RP/RP^ at 52 weeks (left) and quantification (right). (d) Representative images of colon length in WT, *Lrrk2*^RP/+^ and *Lrrk2*^RP/RP^ at 52 weeks (left) and quantification (right). (e) Behavioral analysis of WT, *Lrrk2*^RP/+^, and *Lrrk2*^RP/RP^ at 52 weeks. Motor performance was evaluated by the beam working test, the hanging-wire grip test, rotarod test and open field test of the total distance traveled. Exploratory behavior was evaluated by open field test of the center time. Gastrointestinal function was evaluated by the defecation time of black feces. Experimental data for (a-e) were obtained from seven independent mice, with similar results obtained. Data are shown as the mean ± SD with *p* values by one-way ANOVA with Tukey’s multiple comparison’s test (a-e). *p < .05, **p < .01, ***p < .001, ****p < .0001. (b) Scale bar, 20 μm.

To investigate whether *E*. *coli* interacts with *LRRK2* risk variants to trigger *LRRK2*-associated sPD, we generated a knock-in mouse model harboring the *Lrrk2* R1628P variant (Supplementary Figure S1a). Firstly, we detected the expression and kinase activity of the LRRK2 protein in the colon tissues of *Lrrk2* R1628P mice. Increased LRRK2 kinase activity was observed in both heterozygous and homozygous *Lrrk2* R1628P (*Lrrk2*^RP/+^, *Lrrk2*^RP/RP^) mice (Figure 2a). Moreover, both the *Lrrk2*^RP/+^ and *Lrrk2*^RP/RP^ mice showed disruption of intestinal epithelial barrier (IEB) integrity, as assessed by reduced expression of E-cadherin and β-catenin in the epithelial layer of the colon, and increased intestinal inflammatory in the fecal samples (Figure 2a-c). In addition, colon length was measured to determine the severity of colitis. We observed a significant decrease in colon length in the *Lrrk2*^RP/+^ and *Lrrk2*^RP/RP^ mice compared to wild-type (WT) mice (Figure 2d). Furthermore, the defecation time was significantly prolonged in *Lrrk2*^RP/+^ mice at 52 weeks and in *Lrrk2*^RP/RP^ mice at 36 weeks, whereas no motor dysfunctions were observed (Figure 2e). Together, these data demonstrated that the *Lrrk2* R1628P variant caused IEB impairment and intestinal dysfunctions.

### E. coli triggers α-syn pathology and PD-like symptoms in Lrrk2 R1628P mice

**Figure 3.**
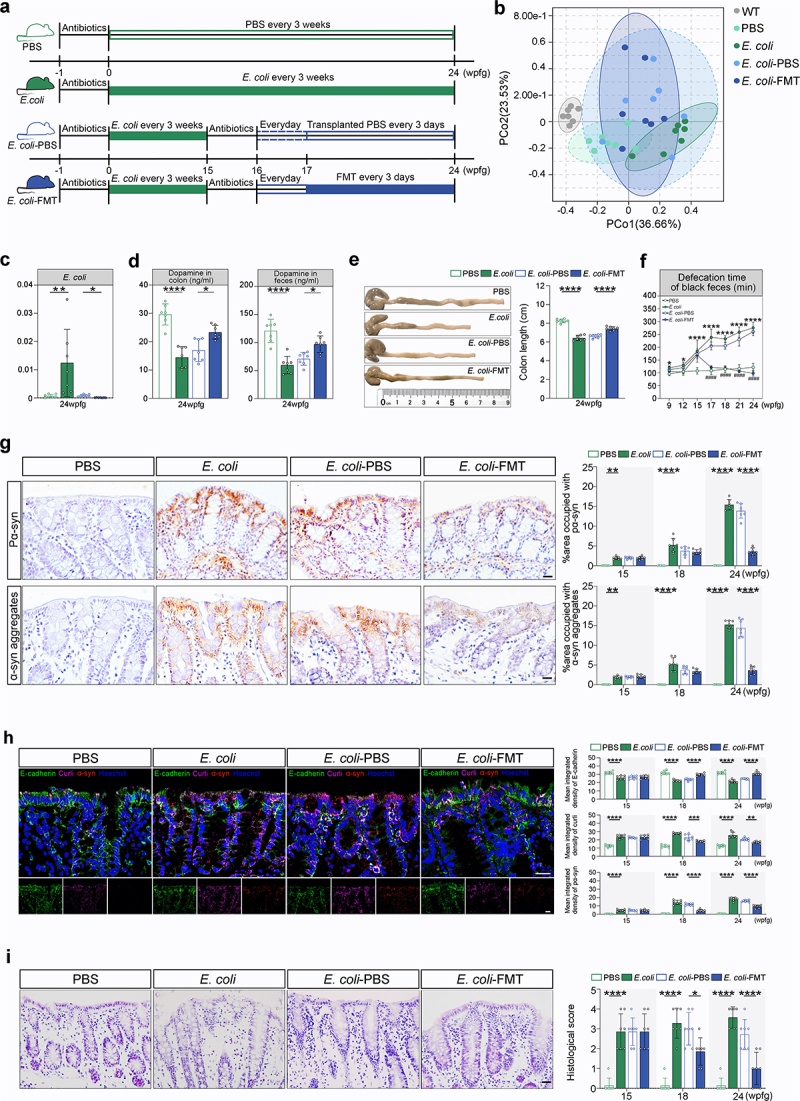
Pathological α-syn is triggered by *E*. *coli* and reversed by FMT in the colon *of Lrrk2*^RP/+^ mice. (a) Timeline schematic of the experimental design showing the procedures of *E*. *coli* administration and FMT in *Lrrk2*^RP/+^. (b) PCoA plot of fecal microbiota data at genus-level in terms of 16S rDNA sequencing among the WT, *Lrrk2*^RP/+^ PBS, *Lrrk2*^RP/+^ *E*. *coli*, *Lrrk2*^RP/+^ *E*. *coli-*PBS, and *Lrrk2*^RP/+^ *E*. *coli-*FMT at 24 wpfg. Ellipses represent a 95% confidence interval. (c) Species-level taxonomic analysis of the intestinal microbiota composition using metagenomic sequencing among the WT, *Lrrk2*^RP/+^ PBS, *Lrrk2*^RP/+^ *E*. *coli*, *Lrrk2*^RP/+^ *E*. *coli-*PBS and *Lrrk2*^RP/+^ *E*. *coli-*FMT at 24 wpfg. (d) HPLC analysis of dopamine levels in colonic and fecal samples from Lrrk2^RP/+^ PBS, *Lrrk2*^RP/+^ *E*. *coli*, *Lrrk2*^RP/+^ *E*. *coli*-PBS and *Lrrk2*^RP/+^ *E*. *coli*-FMT at 24 wpfg. (e) Representative images of colon length in *Lrrk2*^RP/+^ PBS, *Lrrk2*^RP/+^ *E*. *coli*, *Lrrk2*^RP/+^ *E*. *coli*-PBS and *Lrrk2*^RP/+^ *E*. *coli*-FMT at 24 wpfg (left), and quantification (right). (f) gastrointestinal function was evaluated by the defecation time of black feces among *Lrrk2*^RP/+^ PBS, *Lrrk2*^RP/+^ *E*. *coli*, *Lrrk2*^RP/+^ *E*. *coli*-PBS and *Lrrk2*^RP/+^ *E*. *coli*-FMT at 9, 12, 15, 17, 18, 21, and 24 wpfg, respectively. (g) Representative immunohistochemical images of pα-syn and α-syn aggregates in the colon of *Lrrk2*^RP/+^ PBS, *Lrrk2*^RP/+^ *E*. *coli*, *Lrrk2*^RP/+^ *E*. *coli*-PBS and *Lrrk2*^RP/+^ *E*. *coli*-FMT at 15, 18, and 24 wpfg, respectively(left). Quantification used the % area occupied with pα-syn and α-syn aggregates (right). (h) Representative images of E-cadherin (green), curli (magenta) and pα-syn (red) in the colon of *Lrrk2*^RP/+^ PBS, *Lrrk2*^RP/+^ *E*. *coli*, *Lrrk2*^RP/+^ *E*. *coli*-PBS and *Lrrk2*^RP/+^ *E*. *coli*-FMT (left). Quantification of E-cadherin, curli and pα-syn fluorescence intensity at 15, 18, and 24 wpfg, respectively (right). (i) Representative images of HE staining in the colon of *Lrrk2*^RP/+^ PBS, *Lrrk2*^RP/+^ *E*. *coli*, *Lrrk2*^RP/+^ *E*. *coli*-PBS and *Lrrk2*^RP/+^ *E*. *coli*-FMT (left). Quantification of the histological scores at 15,18 and 24 wpfg, respectively(right). Experimental data for (b-i) were obtained from seven independent mice, with similar results obtained. Data are analyzed by PERMANOVA (b), Kruskal-Wallis test with two-stage linear step-up procedure of Benjamini, Krieger and Yekutieli multiple comparison’s test (c), one-way (c-e) or two-way (f-i) ANOVA with Tukey’s multiple comparison’s test. *p < .05, **p < .01, ***p < .001, ****p < .0001. Scale bar, 20 μm (g-i).

Having established that *Lrrk2* R1628P transgenic mice exhibited intestinal dysfunctions, it is critical to determine whether *E. coli*, acting as an environmental factor, interacts with *LRRK2* risk variants to trigger the pathogenesis of *LRRK2*-associated sPD. For this, *Lrrk2*^RP/+^ and *Lrrk2*^RP/RP^ mice were orally gavaged with *E*. *coli* to create an intestinal *E*. *coli* infection model (*Lrrk2*^RP/+^ *E*. *coli* and *Lrrk2*^RP/RP^ *E*. *coli*).^28^ Control mice were administered with phosphate-buffered saline (PBS) (*Lrrk2*^RP/+^ PBS and *Lrrk2*^RP/RP^ PBS) (Figure 3a and Supplementary Figure S1b). In parallel,a species-level taxonomic analysis of the intestinal microbiota composition using metagenomic sequencing showed that the relative abundance of *E*. *coli* was obviously increased in *Lrrk2*^RP/+^ *E*. *coli* compared to *Lrrk2*^RP/+^ PBS (Figure 3c). Next, the morphology of the colon tissues and the extent of inflammatory cell infiltration were evaluated. We found more considerable damage to the epithelial cells with severe loss of goblet cells and multiple mucosal ulcerations in the colonic mucosal layers of *E*. *coli*-treated *Lrrk2* R1628P mice (Figure 3i and Supplementary Figure S1c). Moreover, *Lrrk2*^RP/+^ *E. coli* mice at 24 weeks post-first gavage (wpfg) and *Lrrk2*^RP/RP^ *E*. *coli* mice at 21 wpfg showed markedly shortened colon lengths (Figure 3e and Supplementary Figure S1d). Curli, the functional amyloid fibers produced by *E*. *coli*,^29^ were obviously increased and widely distributed throughout the entire colonic mucosal layer of *E*. *coli*-treated *Lrrk2* R1628P mice, even infiltrating into the submucosal layer (Figure 3h and Supplementary Figure S1e). Furthermore, we observed a significantly prolonged defecation time in *E*. *coli*-treated *Lrrk2* R1628P mice (Figure 3f and Supplementary Figure S1j).

**Figure 4.**
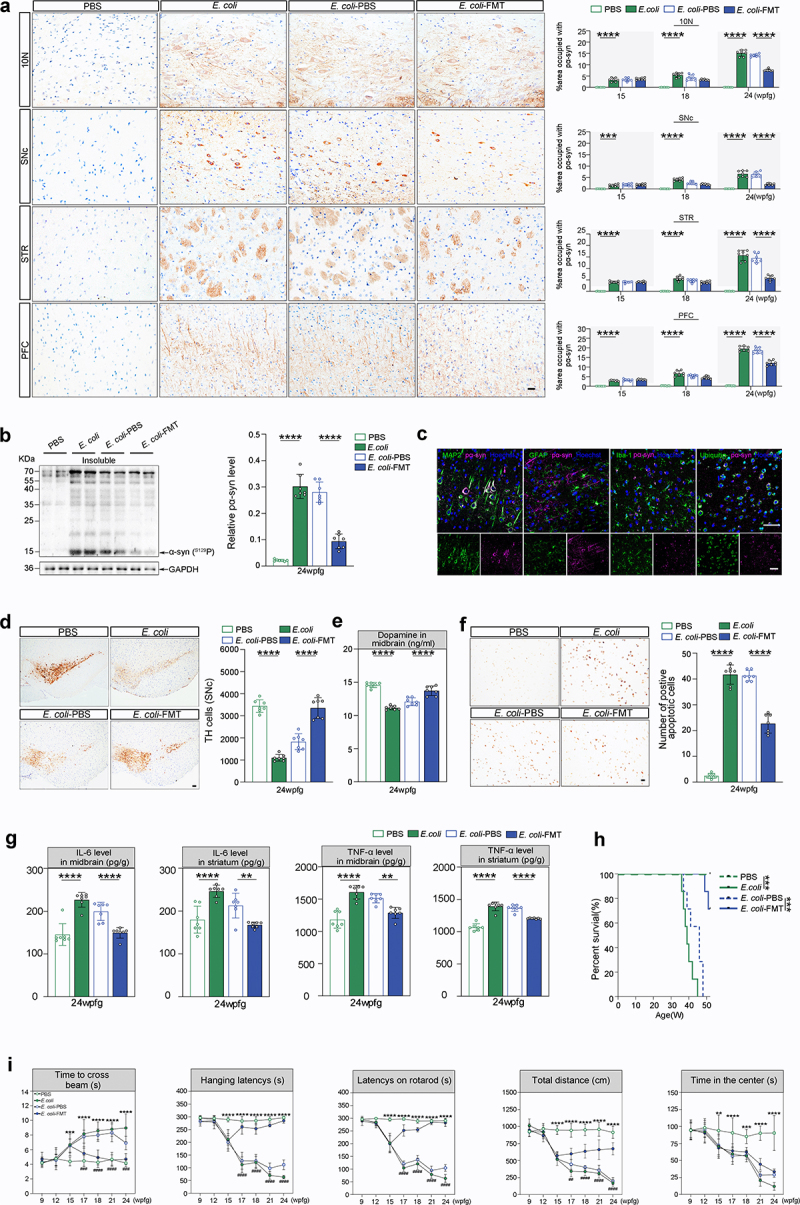
PD-like phenotypes are triggered by *E*. *coli* and reversed by FMT in *Lrrk2*^RP/+^ mice brain. (a) Representative immunohistochemical images of pα-syn in the 10N, SNc, STR, and PFC of *Lrrk2*^RP/+^ PBS, *Lrrk2*^RP/+^ *E*. *coli*, and *Lrrk2*^RP/+^ *E*. *coli*-FMT (left), respectively. Quantification used the % area occupied with pα-syn at 15, 18 and 24 wpfg, respectively. (b) Representative immunoblot images of pS129 α-syn in the insoluble fractions of the midbrain in *Lrrk2*^RP/+^ PBS, *Lrrk2*^RP/+^ *E*. *coli*, *Lrrk2*^RP/+^ *E*. *coli*-PBS, and *Lrrk2*^RP/+^ *E*. *coli*-FMT at 24 wpfg (left) and quantification (right). (c) Representative images of pα-syn (magenta) with MAP2 (green), GFAP (green), iba-1 (green), or ubiquitin (green) of *Lrrk2*^RP/+^ *E*. *coli* at 24 wpfg. Co-immunolabeling is represented by the signal in white. Cell nuclei were counterstained with Hoechst. (d) Representative immunohistochemical images of TH^+^ neurons in SNc in *Lrrk2*^RP/+^ PBS, *Lrrk2*^RP/+^ *E*. *coli*, *Lrrk2*^RP/+^ *E*. *coli*-PBS and *Lrrk2*^RP/+^ *E*. *coli*-FMT (left). Quantification of TH immunoreactivity from mice at 24 wpfg, respectively (right). (e) HPLC analysis of dopamine levels in midbrain of *Lrrk2*^RP/+^ PBS, *Lrrk2*^RP/+^ *E*. *coli*, *Lrrk2*^RP/+^ *E*. *coli*-PBS and *Lrrk2*^RP/+^ *E*. *coli*-FMT at 24 wpfg. (f) Representative TUNEL staining for cell apoptosis in the SNc of *Lrrk2*^RP/+^ PBS, *Lrrk2*^RP/+^ *E*. *coli*, *Lrrk2*^RP/+^ *E*. *coli*-PBS and *Lrrk2*^RP/+^ *E*. *coli*-FMT at 24 wpfg (left) and quantification (right). (g) ELISA analysis for IL-6 and TNF-α level in midbrain and striatum of Lrrk2^RP/+^ PBS, *Lrrk2*^RP/+^ *E*. *coli*, *Lrrk2*^RP/+^ *E*. *coli*-PBS and *Lrrk2*^RP/+^ *E*. *coli*-FMT at 24 wpfg. (h) Kaplan-Meier survival curves of *Lrrk2*^RP/+^ PBS, *Lrrk2*^RP/+^ *E*. *coli*, *Lrrk2*^RP/+^ *E*. *coli*-PBS and *Lrrk2*^RP/+^ *E*. *coli*-FMT. (i) Behavioral analysis of *Lrrk2*^RP/+^ PBS, *Lrrk2*^RP/+^ *E*. *coli*, *Lrrk2*^RP/+^ *E. coli*-PBS and *Lrrk2*^RP/+^ *E*. *coli*-FMT at 9, 12, 15, 17,18, 21, and 24 wpfg, respectively. Experimental data for (a-i) were obtained from seven independent mice, with similar results obtained. Data are shown as the mean ± SD with *p* values by two-way (a, i) or one-way (b-g) ANOVA with Tukey’s multiple comparison’s test and log-rank (mantel-cox) test (h). *p < .05, **p < .01, ***p < .001, ****p < .0001(*Lrrk2*^RP/+^ PBS versus *Lrrk2*^RP/+^ *E*. *coli*) (up); ^#^*p* < .05, ^##^*p* < .01, ^###^*p* < .001, ^####^*p* < .0001 (*Lrrk2*^RP/+^ *E*. *coli*-PBS versus *Lrrk2*^RP/+^ *E*. *coli*-FMT) (down) Scale bar, 20 μm (a, c and f), 25 μm (d).

Amazingly, sparsely distributed pathological α-syn aggregates were detected in the colonic mucosal epithelium of both *Lrrk2*^RP/+^ *E*. *coli* and *Lrrk2*^RP/RP^ *E*. *coli* mice (Figure 3g,h and Supplementary Figure S1f, S2). Subsequently, pSer129 α-syn (pα-syn) immunostaining was used to monitor the progression of pathological α-syn from the gut to the brain. As expected, pathological α-syn was also gradually observed in the dorsal motor nucleus of vagus (10N), the substantia nigra pars compacta (SNc), the striatum (STR), and the prefrontal cortex (PFC) (Figure 4a and Supplementary Figure S1f). However, no pathological α-syn aggregates were detected in either *Lrrk2*^RP/+^ PBS or *Lrrk2*^RP/RP^ PBS mice by the end of the study (Figures 3g,h, 4a and Supplementary Figure S1f). Immunofluorescence staining confirmed that pα-syn was ubiquitin-positive in neurons and partially colocalized with ionized calcium binding adapter molecule 1 (Iba-1) or glial fibrillary acidic protein (GFAP) (Figure 4c). In addition, WB analysis showed that the level of insoluble α-syn in brain lysates was significantly increased in *E*. *coli*-treated *Lrrk2* R1628P mice (Figure 4b).

The median survival times for *Lrrk2*^RP/+^ *E*. *coli* and *Lrrk2*^RP/RP^ *E*. *coli* mice were 39 weeks and 35 weeks, respectively (Figure 4h; Supplementary Figure S1i). The PD-like motor and nonmotor dysfunctions^7^, including balance impairment, motor coordination deficits, muscle strength reduction, anxiety, and constipation were exhibited in *Lrrk2*^RP/+^ *E*. *coli* at 15 wpfg and in *Lrrk2*^RP/RP^ *E*. *coli* at 12 wpfg, respectively (Figure 4i; Supplementary Figure S1j). In parallel, unbiased stereological counts^30^ of tyrosine hydroxylase (TH)-positive neurons (TH^+^) in the SNc revealed significant reduction in the number of TH^+^ in *E*. *coli*-treated *Lrrk2* R1628P mice, which was accompanied by markedly reduced dopamine levels in the midbrain (Figure 4d, e; Supplementary Figure S1h). Moreover, decreased levels of dopamine were also detected in colon and fecal samples of *Lrrk2*^RP/+^ *E*. *coli* and *Lrrk2*^RP/RP^ *E*. *coli* mice (Figure 3d; Supplementary Figure S1h). However, no loss of TH^+^neurons or PD-like symptoms were detected in PBS-treated *Lrrk2* R1628P mice (Figures 3d, 4d, e; and Supplementary Figure S1g, h, j). Furthermore, we found that increased numbers of apoptotic cells evaluated by TUNEL staining were observed in SNc of *Lrrk2*^RP/+^ *E*. *coli* compared to *Lrrk2*^RP/+^ PBS mice (Figure 4f). Besides, levels of pro-inflammatory cytokines, interleukin-6 (IL-6) and tumor necrosis factor-α (TNF-α) measured by ELISA were increased in the midbrain and striatum of *Lrrk2*^RP/+^ *E*. *coli* mice compared to *Lrrk2*^RP/+^ PBS mice, respectively (Figure 4g). Taking together, pathologicalα-syn aggregation and propagation, increased cell apoptosis, and inflammatory reaction may contribute to the dopaminergic neuronal loss in *E*. *coli*-treated *Lrrk2* R1628P mice.

Moreover, to explore whether *E*. *coli* is a specific environmental factor to interact with *LRRK2* risk variants to trigger PD-like pathological alterations, *Lrrk2*^RP/+^ mice were orally gavaged with *K. pneumoniae*, *Proteus mirabilis* (*P*. *mirabilis*), and dextran sodium sulfate (DSS) (SupplementaryFigure S3a)^31,32^. hese three mice displayed colonic mucosal damage and inflammation (Supplementary Figure S4c, d). However, no α-syn pathology was detected in the intestinal tract of the three groups at 24 wpfg (Supplementary Figure S4e). In addition, to study whether the curli produced by *E*. *coli* is essential for α-syn aggregates formation in the intestinal tract, *Lrrk2*^RP/+^ were orally gavaged with curli-deficient mutant *E*. *coli* (*Lrrk2*^RP/+^ mut curli *E*. *coli*), which was unable to produce normal curli (Supplementary Figure S4a).^33^ The results showed that there were no pathological α-syn detected in the intestinal tract of *Lrrk2*^*RP/+*^ mut curli *E*. *coli* at 24 wpfg (Supplementary Figure S4e). These results demonstrated that *E*. *coli* might trigger PD-like progression in *Lrrk2* transgenic mice through curli-induced pathological α-syn aggregation. Moreover, to test the contribution of *LRRK2* risk variants to the progression of *E*. *coli* induced pathological α-syn aggregation. WT (C57BL/6) and *Snca* A53T transgenic mice were orally gavaged with *E*. *coli* (WT *E*. *coli*, *Snca* A53T *E*. *coli*) for 24 weeks (Supplementary Figure S4a). We found that there was no damage to the mucosal architecture, and no pα-syn was detected in the colon tissue of both WT *E*. *coli* (Supplementary Figure S4b) and *Snca* A53T *E*. *coli* mice (Supplementary Figure S4c, d, e). Collectively, these results indicated that *E*. *coli* could specifically drive the progression of sPD with *LRRK2* risk variants, which further suggested that the specific genetic risk variants and their corresponding environmental factors mightsynergistically contribute to neurodegenerative diseases.

### E. coli-derived EVs induce α-syn aggregation in Lrrk2 R1628P mice

**Figure 5.**
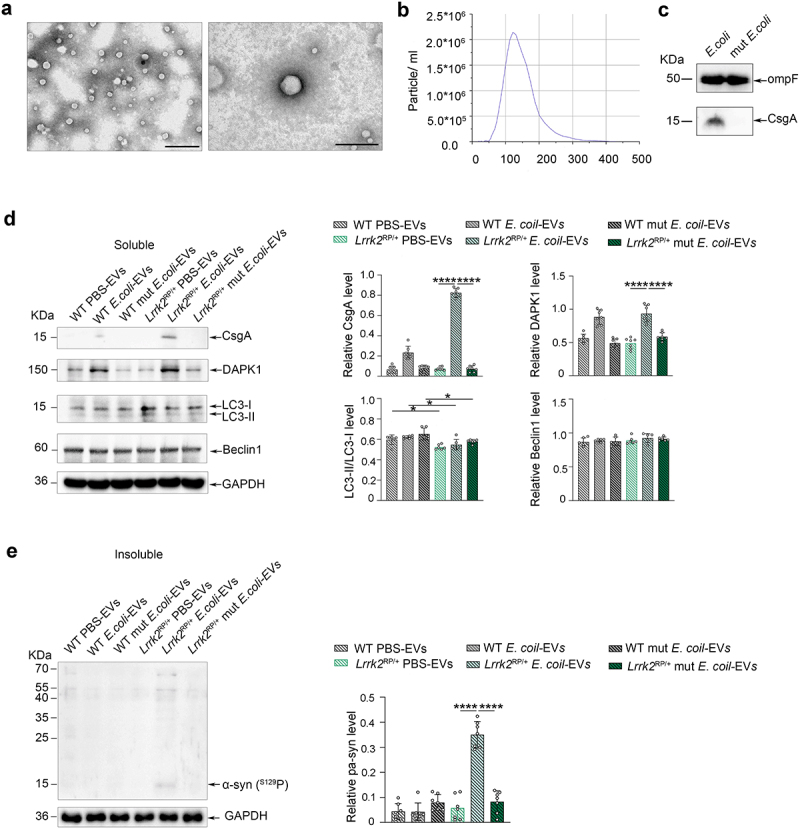
Co-occurrence of increased α-syn phosphorylation and autophagic impairment triggered α-syn aggregation in *Lrrk2* R1628P mice. (a) characterization of intestinal *E*. *coli*-derived extracellular vesicles (EVs). Transmission electron microscopy image of *E*. *coli*-derived EVs at different magnifications. (b) size distribution of *E*. *coli*-derived EVs according to diameter as determined by nanoparticle tracking analysis. (c) Representative immunoblot images of OmpF and CsgA levels in *E*. *coli*-derived EVs and mut *E*. *coli*-derived EVs. (d) Representative immunoblot images of CsgA, DAPK1, LC3-II/LC3-I, Beclin1 levels in the soluble fractions of colon in *Lrrk2*^RP/+^ (*Lrrk2*^RP/+^ *E*. *coli*-EVs, *Lrrk2*^RP/+^ mut *E*. *coli*-EVs, *Lrrk2*^RP/+^ PBS-EVs) and WT (WT *E*. *coli*-EVs, WT mut *E*. *coli*-EVs, WT PBS-EVs) at 8 weeks after injection (left) and quantification (right). (e) Representative immunoblot images of pS129 α-syn in the insoluble fractions of the colons in Lrrk2^RP/+^ *E*. *coli*-EVs, Lrrk2^RP/+^ mut *E*. *coli*-EVs, Lrrk2^RP/+^ PBS-EVs, WT *E*. *coli*-EVs, WT mut *E*. *coli*-EVs, and WT PBS-EVs mice at 8 weeks after injection (left) and quantification (right). Experimental data for (c-e) were obtained from seven independent mice, with similar results obtained. Data are shown as the mean ± SD with *p* values by one-way ANOVA with Tukey’s multiple comparison’s test (a-e). *p < .05, **p < .01, ***p < .001, ****p < .0001. Scale bar, left 1.0 μm, right 200 nm (a).

It is well documented that bacteria communicate with the host cells and other bacteria through the release of membrane vesicles known as EVs. *E*. *coli*-derived EVs may harbor pathogen-associated molecular patterns and involve in the pathogenesis of diseases. To investigate the mechanism behind the *E*. *coli*-induced α-syn aggregation in *Lrrk2* R1628P mice, we purified and characterized the EVs derived from *E*. *coli*. The EVs were extracted from the cultured supernatant of *E*. *coli*,^34^ and transmission electron microscopy was used to characterize their morphology (Figure 5a). The majority of the purified EVs exhibited a smooth, saucer-like morphology withdiameters ranging from 100–170 nm (Figure 5b). WB analysis showed that both *E*. *coli*-derived EVs and mut *E*. *coli*-derived EVs contained OmpF (Figure 5c), a marker used to quantify *E*. *coli*-derived EVs.^35^ Notably, curli was only detected in *E. coli-*derived EVs, as indicated by the measurement of total CsgA levels (Figure 5c). To verify whether α-syn phosphorylation related protein kinases are involved in *E*. *coli*-induced α-syn aggregation, a total of 25 µg purified EVs derived from *E*. *coli*, mut *E*. *coli,* and an equivalent dose of PBS were injected into the colon of *Lrrk2*^*RP/+*^ mice (*Lrrk2*^RP/+^ *E*. *coli*-EVs, *Lrrk2*^RP/+^ mut *E*. *coli*-EVs, *Lrrk2*^*RP/+*^ PBS-EVs) and WT mice (WT *E*. *coli*-EVs, WT mut *E*. *coli*-EVs, WT PBS-EVs). Interestingly, curli was enriched in the purified EVs from *Lrrk2*^RP/+^ *E*. *coli*-EVs. However, no curli was detected in the other mouse models (Figure 5d). Surprisingly, we only detected insoluble α-syn expression in the colon of *Lrrk2*^RP/+^ *E*. *coli*-EVs mice at 8 weeks after injection (Figure 5e). Moreover, only DAPK1, a regulator of toxic α-syn aggregation,^36^ was upregulated in the colons of *Lrrk2*^RP/+^ *E*. *coli*-EVs and WT *E*. *coli*-EVs mice (Figure 5d). Furthermore, we also detected inhibition of autophagic degradation measured by the decreased expression level of the LC3-II/LC3-I ratio^37^, a marker of autophagy, in *Lrrk2*^*RP/+*^ PBS-EVs, *Lrrk2*^RP/+^ *E*. *coli*-EVs, and *Lrrk2*^RP/+^ mut *E*. *coli*-EVs mice.

Collectively, these results suggested that *E*. *coli*-derived EVs delivered a large number of curli into the colonic epithelial cells to boost DAPK1 expression in the colon of *Lrrk2* R1628P mice. The combined effect of DAPK1 upregulation and autophagic inhibition induced by the *LRRK2* R1628P variant in the colon resulted in an imbalance in the phosphorylation and degradation of α-syn, eventually causing α-syn aggregation in *E*. *coli* treated *Lrrk2* R1628P mice.

### FMT reverses PD progression in E. coli-treated Lrrk2 R1628P mice

To further prove the involvement of *E*. *coli* drived-intestinal microbiota dysbiosis in *LRRK2*-associated sPD pathogenesis and to evaluate the potential protective effects of FMT treatment on *E*. *coli*-treated *Lrrk2* R1628P mice, we transplanted the fecal microbiota from healthy donor mice (WT mice, 12 weeks of age) into a random sample of *Lrrk2*^RP/+^ *E*. *coli* and *Lrrk2*^RP/RP^ *E*. *coli* mice at 15 wpfg and 12 wpfg (*Lrrk2*^RP/+^ *E*. *coli*-FMT and *Lrrk2*^RP/RP^ *E*. *coli*-FMT), respectively. The control mice were transplanted with PBS (*Lrrk2*^RP/+^ *E*. *coli*-PBS and *Lrrk2*^RP/RP^ *E*. *coli*-PBS) at the same time points (Figure 3a and Supplementary Figure S1b). As illustrated in Figure 3b, PCoA revealed that the intestinal microbiota composition of *Lrrk2*^RP/+^ *E*. *coli*-FMT mice was closer to that of WT PBS mice compared with *Lrrk2*^RP/+^ *E*. *coli* mice. Specifically, in comparison with the *Lrrk2*^RP/+^ *E*. *coli*-PBS mice, the relative abundance of *E*. *coli* was significantly decreased in *Lrrk2*^RP/+^ *E*. *coli*-FMT mice (Figure 3c). Collectively, these data provide evidence that FMT alleviates the microbiota dysbiosis induced by *E*. *coli* administration.

Meanwhile, FMT treatment exerted a profound improvement on the intestinal pathological changes of *E*. *coli*-treated *Lrrk2* R1628P mice. We observed an obvious increase in E-cadherin expression and a decrease in curli expression in the colonic mucosa of *Lrrk2*^RP/+^ *E. coli*-FMT and *Lrrk2*^RP/RP^ *E*. *coli*-FMT mice compared to *Lrrk2*^RP/+^ *E*. *coli*-PBS and *Lrrk2*^RP/RP^ *E*. *coli*-PBS mice at 2 weeks after the first FMT, respectively (Figure 3h and Supplementary Figure S1e). Surprisingly, pathological α-syn was markedly reduced in the colonic mucosa epithelial cells and goblet cells of *Lrrk2*^RP/+^ *E*. *coli*-FMT and *Lrrk2*^RP/RP^ *E*. *coli*-FMT mice (Figure 3g, h and Supplementary Figure S1f). Moreover, intestinal inflammation was attenuated with the improvement of mucosal barrier integrity and the length of colon (Figure 3e, 3 and Supplementary Figure S1c, d). Surprisingly, pathological α-syn aggregates were reduced to some extent in the 10N, SNc, STR and PFC at 8 weeks after the first FMT (Figure 4a and Supplementary Figure S1f). In parallel, WB analysis confirmed a decrease in pα-syn expression in brain tissues (Figure 4b). In addition, FMT significantly improved the balance ability, motor coordination, and muscle strength in *Lrrk2*^RP/+^ *E*. *coli*-FMT and *Lrrk2*^RP/RP^ *E*. *coli*-FMT mice (Figure 4i and Supplementary Figure S1j). Likewise, the defecation time was obviously shortened in *Lrrk2*^RP/+^ *E*. *coli*-FMT and *Lrrk2*^RP/RP^ *E*. *coli*-FMT mice at 1 week after the first FMT treatment (Figure 3f and Supplementary Figure S1j). Additionally, FMT restored the levels of TH expression in the SNc evaluated by immunohistochemistry (Figure 4d and Supplementary Figure S1g), and the dopamine levels were significantly increased in midbrain, colon, and fecal samples evaluated by HPLC at 8 weeks after the first FMT in *Lrrk2*^RP/+^ *E*. *coli*-FMT and *Lrrk2*^RP/RP^ *E*. *coli*-FMT mice (Figures 3d, 4e and Supplementary Figure S1h). Moreover, the numbers of apoptotic cells were decreased in SNc of *Lrrk2*^RP/+^ *E*. *coli*-FMT mice compared to *Lrrk2*^RP/+^ *E*. *coli*-PBS mice (Figure 4f). Meanwhile, reduced IL-6 and TNF-α levels were detected in the midbrain and striatum of *Lrrk2*^RP/+^ *E*. *coli*-FMT mice (Figure 4g). Notably, FMT significantly and substantially prolonged the lifespan of mice, as compared with *Lrrk2*^RP/+^ *E*. *coli*-PBS and *Lrrk2*^RP/RP^ *E*. *coli*-PBS mice (Figure 4h and Supplementary Figure S1i). Taken together, these findings suggested that restoring the balance of the intestinal microbiota composition could alleviate, and potentially reverse, the *E*. *coli*-induced intestinal pathological changes and PD-like phenotypes in *Lrrk2* R1628P mice.

## Discussion

PD is a multifactorial neurodegenerative disease and gene-environment interactions are likely to explain the pathogenesis of sPD in carriers of the risk-increasing genetic variants related to sPD^38^. In the past decade, considerable progress has been made in the field of sPD related risk-increasing genetic variants^39^. Meanwhile, a series of preceding efforts from epidemiological, in vitro, and in vivo studies have identified that herbicides, pesticides, head trauma and heavy metals are considered to be major environmental risk factors of sPD.^40^ However, diverse genetic backgrounds may result in different effects of genetic susceptibility.^41^ Therefore, there should be a one-to-one correspondence between the gene-environment interaction and the understanding of mechanisms of specific gene-environment interaction pattern may open the door to the early recognition, treatment, and even prevention of sPD.

*LRRK2* is one of the most common sPD risk gene in Asians, and coincidentally shared risk allele for CD.^11,42^ However, the corresponding environmental factors of *LRRK2* risk variants contributing to α-syn aggregation remain unknown in sPD. Both of these two diseases are chronic progressive diseases which may share common pathophysiological links.^18^ Increasing studies have noted that intestinal microbiota dysbiosis is likely to sustain mucosal inflammation and hasbeen linked to increased risk for sPD and CD.^43,44^ However, few studies have characterized the intestinal microbiota composition of patients with *LRRK2*-associated sPD. In our study, noticeable differences in the diversity and composition of the intestinal microbiome were detected between *LRRK2+*/sPD+ and *LRRK2+*/sPD- participants. Markedly, the relative abundance of *E*. *coli* in the *LRRK2+*/sPD+ group was almost seven times higher than that in the *LRRK2+*/sPD- group. Recent evidence suggests that *LRRK2* may act as a central regulator in inflammation and pathogen defense in a cell-type/organ-specific manner.^45,46^

In our study, the *LRRK2* R1628P variant caused IEB impairment and intestinal dysfunctions in *Lrrk2* R1628P mice. Furthermore, the colonization of the oral gavage with *E*. *coli*, providing an *E*. *coli*-rich environment in the intestinal tract, directly triggered the core pathology of α-syn aggregates as well as motor deficits in *Lrrk2* R1628P mice. Our results showed that *E*. *coli* administration induced a large number of curli infiltrating into the colonic mucosal layer without affecting curli production process of *E*. *coli*. The disruption of IEB induced by *Lrrk2* R1628P variant may increase the translocation of *E*. *coli* and *E*. *coli*-produced curli, potentially contribute to the pathological α-syn accumulation and propagation.^47^ Meanwhile, the pathological α-syn accumulation within enteric nervous system may decrease the level of dopamine in colon, which could contribute to the GI dysfunction. Besides, the spreading of pathological α-syn from the gut to the brain through the vagus nerve could exacerbate GI dysfunction of *Lrrk2* R1628P mice. In addition, we also performed the truncal vagotomy (TV) before the administration of *E. coli* in *Lrrk2*^RP/+^ and before the injection of α-syn preformed fibrils (PFFs) in WT mice (WT-PFFs-TV). We found that there is no pathological α-syn aggregations in the dorsal motor nucleus of 10N, SNc, STR and PFC at 14 weeks post-injection (wpi) in WT-PFFs-TV, respectively. However, with the administration of *E. coli*, most of the TV-treated *Lrrk2*^RP/+^ mice had a very high mortality rate at 6 wpfg (84%). Consistent with previous research,^48,49^ α-syn pathology could spread from the gut to brain via the vagus nerve in our mouse models.

However, neither curli-deficient *E*. *coli* nor other bacteria, such as *K. pneumoniae* and *P*. *mirabilis*, could induce α-syn aggregation in the intestinal tract. Moreover, we investigated whether *E. coli* from PD patients could induce PD-like progression and gut inflammation in *Lrrk2* R1628P mice. *E. coli* strains (NRG 857C and HM427) were isolated from the fecal suspension of sPD patients, and were regularly gavaged together to *Lrrk2 t*ransgenic mice (*Lrrk2*^RP/+^ PD-*E*. *coli*, *Lrrk2*^RP/RP^ PD-*E*. *coli*) every 3 weeks for 10 weeks. However, we have not observed pathological α-syn accumulation in the colon tissue of the mice at 10wpfg (Supplementary Figure S3a). A possible reason for this might be the short observation period of these mouse models. Besides, taking into account the fundamental species differences between humans and rodents, we also investigated whether the *Citrobacter rodentium* (CR), a natural murine intestinal pathogen that is used as a model of human enteropathogenic *E*. *coli* infection,^50^ could trigger α-syn pathology in *Lrrk2* transgenic mice. We found the damaged colonic mucosa and sparsely distributed pathological α-syn in CR-treated *Lrrk2* transgenic mice (Supplementary Figure S3b). Additionally, *E*. *coli* could not generate pα-syn aggregates in the intestinal tract of WT and *Snca* A53T transgenic mice. In summary, our study indicates that a one-to-one correspondence exists between *LRRK2* risk variants and *E*. *coli* in the gene-environment interaction underlying *LRRK2*-associated sPD.

EVs can be released by both prokaryotes and eukaryotes, and contain constituents including cell-surface proteins, DNA, RNA, lipids, and metabolites.^51^ EVs play a crucial role in regulating intercellular communication and maintaining cellular homeostasis.^52^ Moreover, curli exists on the surface of *E*. *coli*,^53^ could enhance *E*. *coli* adherence.^54^ Recent studies reported that curli could serve as a template for α-syn misfolding,^28,33^ however, the exact mechanism remains unknown. In our study, the administration of *E*. *coli* to *Lrrk2* R1628P mice induced an increased and wide distribution of curli throughout the colonic wall via the disruption of epithelial tight junctions. Then, we demonstrated that curli was delivered into the colonic mucosal epithelial cells through *E*. *coli*-derived EVs to upregulate DAPK1 expression, accompanied by *LRRK2* R1628P variant-induced autophagic impairment, eventually triggering α-syn aggregation.

Our study also suggested that *E*. *coli* administration could induce dopaminergic neuronal loss, which could be restored by FMT treatment in *Lrrk2* R1628P mice via inhibiting cell apoptosis and inflammation responses by improved intestinal microbiota composition. The imbalance of the intestinal microbiota composition may promote a pro-inflammatory environment and influence disease susceptibility.^55^ Further work is needed to clarify the related molecular mechanisms of *E*. *coli* -induced inflammatory responses and cell apoptosis in *LRRK2*-associated sPD.

Targeted at intestinal microbiota dysbiosis, FMT has proven potential in the treatment of *Clostridium difficile* infections and other disorders, such as irritable bowel syndrome.^56,57^ Several studies have reported the benefits of FMT in animal models of PD.^58,59^ In line with these finding, our study found FMT significantly improved the balance ability, motor coordination, muscle strength, and obviously shorten the defecation time of *E*. *coli*-treated *Lrrk2* R1628P mice by restoring the balance of the intestinal microbiota composition. Correspondingly, elevated dopamine contents in both brain and intestine tissues were detected after FMT. It has been established that peripheral production of neurotransmitters by the intestinal microbiota might alter brain chemistry and influence the clinical manifestations.^59^ These findings may explain the improvement in both motor symptoms and nonmotor symptoms of *E*. *coli*-treated *Lrrk2* R1628P mice after FMT treatment. Further studies involving an analysis of the metabolomics of intestinal bacteria will allow us to collect detailed information to identify the exact role of FMT treatment in our mouse models. Most strikingly, reduced α-syn pathology was also detected in intestine and brain tissues of *E*. *coli*-treated *Lrrk2* R1628P mice after FMT. In conclusion, these findings provide powerful evidence for the potential of FMT in reversing the progression of *LRRK2*-associated sPD. Clinically, a small number of case reports and single-arm clinical trials reported that FMT via colonoscopy can relieve the non-motor symptoms with acceptable safety in sPD patients.^60^ So far, an objective assessment of the therapeutic effect of FMT in *LRRK2*-associated sPD has not been performed. Future research will focus on evaluating the therapeutic efficacy of FMT in the treatment of patients with *LRRK2*-associated sPD.

In conclusion, our studies provide evidence of altered composition of the intestinal microbiota, especially the markedly increase in the abundance of *E*. *coli* in patients with *LRRK2*-associated sPD. *E*. *coli*-derived intestinal microbiota dysbiosis directly triggers pathological α-syn aggregation in the colon, which is then transmitted to the brain through the gut-brain axis, accompanied with PD-like symptoms (Supplementary Figure S6). The co-occurrence of increased phosphorylation of α-syn induced by curli from *E*. *coli -*derived EVs and *Lrrk2* variant-induced inhibition of α-syn autophagic degradation eventually co-triggered α-syn aggregation in vivo. FMT effectively ameliorated both motor and non-motor symptoms and markedly reduced α-syn pathology in *Lrrk2* R1628P mice. Our findings highlight a novel gene-environment interaction pattern between *LRRK2* risk variants and *E. coli* in pathological α-syn initiation and the pathogenesis of *LRRK2*-associated sPD, providing new targets for interfering with the onset and progression of the disease.

## Methods

### Ethics statement

The human study about the microbiome analysis was approved by the Institutional Ethics Committees of the First Affiliated Hospital of Zhengzhou University (ZZU) (2021-KY-0385-002). The mice experiments were performed with the ethical approval by the Institutional Animal Care and Use Committee (IACUC) of the First Affiliated Hospital of ZZU.

### Human subjects

We randomly selected patients with sPD and age- and sex- matched HCs from the Henan Idiopathic Parkinson’s Disease and Parkinsonism Multicenter Database from Jan, 2015 to Mar, 2021. In total, 51 *LRRK2*+/sPD+, 21 *LRRK2*+/sPD- and 30 HCs were enrolled in this study. For all individuals, the demographic information was collected on the day of admission.

Inclusion and exclusion criteria Inclusion criteria for patients: should meet the clinical PD diagnosis (MDS-PD criteria) and Hoehn & Yahr stage of 1–3 in OFF.^61^ All subjects gave informed consent prior to study participation. Exclusion criteria included diabetes, anesthetic allergy, antibiotic allergy, psychiatric illness, dementia or MMSE < 25, infection with HIV, syphilis, hepatitis B or C, abdominal or anorectal surgery or trauma history, gastrointestinal or respiratory tract infection, gastrointestinal diseases (ulcerative colitis, irritable bowel syndrome, acid reflux, etc), the medicine (e.g., antibiotics, probiotics or immunosuppressive agents) used within 3 months and without *LRRK2* variant (G2385R, R1628P). All subjects gave informed consent prior to study participation.

Inclusion criteria for HCs age- and sex- matched healthy individuals. Exclusion criteria included abnormal results on a serologic or stool screening, tested positive for *Helicobacter pylori*, therapy with proton pump inhibitors, antibiotics or probiotics use within 3 months, declined to participate, BMI > 30 or < 18, mental health condition, unhealthy lifestyle habits, gastrointestinal disorders, infection with HIV, syphilis, hepatitis B or C.

### Mice

*Lrrk2* R1628P knock-in mice were constructed by Beijing Biocytogen Co., Ltd (Beijing, China). Cas9 mRNA, sgRNA, and oligo donor cocktails were co-microinjected into the cytoplasm of zygotes derived from a C57BL/6J mouse strain. *Lrrk2* R1628P knock-in mice carry one nucleotide substitution mutation (c.4883 G > C) in exon 34 of the *Lrrk2* gene that results in the R1628P amino acid substitution. The chimeras were crossbred with C57BL/6J breeders to generate mice containing the targeted locus through germline transmission (Figure S1A). We obtained heterozygous *Snca* A53T transgenic mice (B6; C3-Tg-Prnp/Snca*A53T/83Vle/J) and WT mice (C57BL/6) from the Shanghai Model Organisms Center, Inc. Mice were housed in the proper humidity (50–60%) and room temperature (23°C, daily temperature difference < 3°C) under 12 h light/dark cycles (lights on at 8:00 am). The mice were allowed free access to commercial rodent food and sterilized tap water. Eight-week-old mice (females and males) were used randomly in this study.

### Microbe strains

CD-associated adherent-invasive *E*. *coli* (AIEC) strain LF82 and mut curli *E*. *coli* (LF82 strain with an isogenic mutant lacking gene encoding the curli biosynthesis machinery) were used in this study.

### Biospecimen collection and preparation

The fresh fecal samples from *LRRK2*+/sPD+, *LRRK2+*/sPD- and HCs were collected at least 20 g in sterile stool collection containers and immediately preserved at −80°C cryogenic freezer until DNA extraction.

### Bacterial administration

*E*. *coli* and mut curli *E*. *coli* were cultivated on lysogeny broth or agar overnight with shaking at 140 rpm and 37°C. *K*. *pneumoniae* (ATCC10031) and *P. mirabilis* (ATCC15146) were grown in nutrient broth overnight with shaking at 140 rpm and 37°C. After centrifugation of the bacterial cultures, pellets were resuspended in sterile PBS and measured by absorbance at 600 nm (Multiskan Microplate Reader, Thermo Fisher Scientific, USA) to confirm the administered colony-forming units (CFU).^62^

### Isolation and characterization of EVs from *E. coli*

Briefly, the *E*. *coli* (LF82) was cultured in lysogeny broth media at 37°C for 24 h. The EVs were extracted and purified by an exosome isolation kit (Umibio, Cat. No: UR52121, China) according to the manufacturer’s instructions. The culture supernatant was collected after 3,000 g and 4°C for 10 minutes, and then mixed with exosome concentration solution (Umibio). The mixtures were vortexed and incubated at 4°C for up to 2 hours and then centrifuged at 10,000 g and 4°C for 60 minutes to precipitate EVs pellets. Pellets of EVs were resuspended in 1×PBS and purified an exosome purification filter at 3,000 g and 4°C for 10 minutes. We measured the particle size and concentration of EVs via nanoparticle tracking analysis (NTA) at Viva Cell Biosciences with Zeta View PMX 110 (Particle Metrix, Meerbusch, Germany) and the corresponding software Zeta View 8.04.02. The ultrastructure and size of EVs were analyzed by transmission electron microscopy (Hitachi, HT7800, Japan). The EV pellet was dissolved in lysis buffer for WB analysis with a marker of *E*. *coli*-derived EVs. The EVs were stored at −80°C immediately after isolation for further processing.

### Colonic injection with EVs from *E. coli*

Mice were anesthetized by intraperitoneal (i.p.) injection of pentobarbital (10% v/v in saline), and kept at a constant body temperature using a conventional heat pad. The colon was injected with 25 µg *E*. *coli*-derived EVs in PBS (2.5 µg/µL, 10 µL total) or equivalent PBS was injected in four different locations. The injection was conducted into the wall of the colon using a 10 µL Hamilton syringe by inserting the needle tip bevel facing up into the colon wall at a 15 angle. After injection, the abdominal muscle/peritoneal layer and skin were sutured separately, and then mice were injected with ketoprofen (2 mg/kg) and placed on a heating pad for recovery.

### Preparation of different mouse models and FMT treatment

Before oral gavage of bacteria, mice were first treated by oral gavage with 200 μL of an antibiotic-cocktail containing 1 g/L ampicillin, 1 g/L neomycin, 0.5 g/L vancomycin, and 1 g/L metronidazole for 3 days followed by a 4-day wash-out period to eliminate antibiotics.^63^ *Lrrk2* ^RP/+^ transgenic mice were regularly administrated 200 μL bacterial suspension (*E*. *coli*, mut curli *E*. *coli*, *K*. *pneumoniae* or *P*. *mirabilis*) (5 × 10^8^ CFU/mL in PBS) by oral gavage after overnight fasting every 3 weeks for 24 weeks. *Lrrk2*^RP/RP^ mice were treated with 200 μL of *E*. *coli* suspension (5 × 10^8^ CFU/mL in PBS) by oral gavage every 3 weeks for 21 weeks. *Snca* A53T transgenic mice were regularly administrated 200 μL of *E*. *coli* suspension (5 × 10^8^ CFU/mL in PBS) by oral gavage after overnight fasting every 3 weeks for 24 weeks. Control mice were treated with an equal volume of PBS at the same time points.^64^

For FMT, fresh feces from healthy donor mice (wild type mice, 3-months old) were collected in sterile tubes every day. Fresh fecal pellets (100 mg) were suspended in 1 mL PBS with 20% glycerol (w/v). After filtration through a 70-μm strainer, the fecal suspension was collected and immediately snap-frozen and transferred for storage at −80°C. After oral gavage with *E*. *coli* in *Lrrk2*^RP/+^ and *Lrrk2*^RP/RP^ mice for 15 weeks and 12 weeks, respectively, antibiotic treatment was performed for 3-day and 4-day wash-out periods to eliminate antibiotics. Then, the *Lrrk2*^RP/+^ *E*. *coli*-FMT and *Lrrk2*^RP/RP^ *E*. *coli*-FMT mice were administered with 100 μL of fecal suspension from healthy donor mice by oral gavage for 7 days. Then, the mice were treated with the fecal suspension every 3 days during the remaining 7 weeks. The control mice were treated with PBS by oral gavage at the same time points.^65^

For DSS treatment, a dose of 2% (w/v in distilled water) DSS (MP Biomedicals) solution was administered to induce experimental colitis by drinking freely for 1 week and then receiving distilled water for the following 2 weeks over a course of 8 cycles.^31^ All animals were followed for 43 weeks after the first antibiotic treatment.

### Behavioral analysis

All the behavioral tests were conducted during the light cycle and independently scored by two investigators in a blinded fashion. Before the behavioral tests, mice were habituated to the dimly lit and sound-shielded room for 60 min. The testing equipment was cleaned with 70% ethanol between animals.

#### Beam walking

The beam walking test was used to examine motor coordination and balance. The beams were 80 cm long, one beam was 0.8 cm wide and the other was 1.6 cm wide, and placed horizontally 50 cm above the floor. Before the formal experiment, 2 daily sessions of 3 trials were performed using the 1.6 cm width beam. Mice were then tested using the beam (0.8 cm width) and allowed to perform in 3 consecutive trials, with a maximum time of 15 s allowed on the beam. As the mice traversed the inclined beam, the times for traversing 50 cm from 3 trials were recorded and averaged.

#### Wire hang test

To evaluate muscle strength, the mouse was placed on a wire mesh, which was gently waved so that the mouse gripped the wire and then inverted. The latency to successfully raise their hind legs to grip the wire was recorded. Latency to fall was recorded with a 5 min cutoff time and recorded for 3 consecutive trials.

#### Rotarod

To assess motor coordination and balance ability, each mouse was tested with the rotarod apparatus (MED-Associates). Mice were first given training on an accelerating rotarod (3 trials, 5 min apart) to ensure that they could remain on the rotarod. In the formal test (1 h later), mice were placed on the rotarod device in accelerating speed mode (increasing from 4 rpm to 40 rpm in 5 min), with a maximum test time of 5 min. The time that had elapsed when the mouse fell from the rotarod was recorded. The process was repeated three times, and the mean was used for analysis.

#### Open field test

The open field test was used to measure general activity, locomotion and anxiety. The mouse was placed in the square open field apparatus (XR-XY1032) and allowed to freely explore for up to 10 min. Spontaneous records and scoring were completed by VisuTrack software (XR-VT101). Locomotion and activity were measured by calculating the total distance traveled and the center area duration time was recorded automatically during the test. Experimenters were blinded to the groups of mice.

#### Gastrointestinal function assessments

The first black stool time was examined with the ink gastric method. After 16 h of fasting, all mice were treated with 10% activated carbon by oral gavage (0.1 mL). The mice were immediately moved into clean, empty individual cages to observe the appearance of black feces and provided water. The time from the oral gavage to the passing of the first dark feces (in min) was recorded. The maximum observation time was 300 min.

### Mouse samples collection and preparation

For fecal samples collection, mice were placed in an empty plastic cage (1 mouse per cage), free of bedding and sterilized with 70% ethanol. A cotton swab was used to rub the abdomen and anus of the mice to stimulate defecation. Fecal samples were immediately collected with sterile forceps, placed in a sterile cryovial and snap-frozen on dry ice, and then stored at −80°C for subsequent experiments. For intestinal inflammatory marker measurements, 4–5 fresh feces pellets were weighed, and mixed with sterile PBS at a concentration of 0.1 g/mL. Samples were ground on ice for 15 min before centrifugation at 5,000 g at 4°C for 10 min, and the supernatants were stored at −80°C until measurements.

For tissue collection, mice were anesthetized by i.p. injection of pentobarbital (10% v/v in saline), and then received transmyocardial perfusion of ice-cold PBS. The colon and brain were rapidly removed. The entire colon was cleaned and the colon lengths of the mice were measured. After clearing gastrointestinal contents, tissues were cut transversely into small pieces, and the colon tissues were used for detection. The tissues were post-fixed in 4% paraformaldehyde solution at 4°C overnight and kept in a mixture of 30% sucrose solution and 4% paraformaldehyde for 24 h, embedded in paraffin and cut into 4 μm thick sections finally. Brain tissues were embedded in the Tissue-Plus OCT compound (Thermo Fisher Scientific, USA) and flash-frozen, sliced (25-μm thick sections) with a cryostat (Leica, Germany) and kept in PBS at 4°C.

### 16S rDNA amplicon sequencing and analysis

In brief, the extracted DNA from the fecal samples of both humans and mice underwent a PCR using 341-Forward and 806-Reverse primers for the V3-V4 variable region of the bacterial 16S rRNA gene.^66^ Purified DNA was sequenced using a NovaSeq platform and 250 bp paired-end reads were generated. Denoise was performed with the DADA2 or deblur module in the QIIME2 software (version QIIME2–202006) to obtain initial ASVs.^67^ The representative sequences (named “features” in QIIME2 nomenclature) were generated, and the redundant and low occurrence (*n* < 5 within all samples) sequences were removed. The absolute abundance of ASVs was normalized using a standard of sequence number corresponding to the sample with the fewest sequences. The 16S rDNA amplicon sequencing data from fecal samples were obtained from Beijing Novogene Biotechnology Co., Ltd, China.

### Shotgun metagenomic DNA sequencing and analysis

In brief, DNA was isolated using the Magnetic Stool DNA Kit (Tiangen Biotech, China) in accordance with the manufacturer’s protocols, which were quantified by Qubit® dsDNA Assay Kit in Qubit® 2.0 Fluorometer (Life Technologies, USA). The shotgun metagenomic sequencing was performed on the Illumina NovoSeq platform, generating on average 20 million reads (~6 Gb) per sample. High-quality reads were screened by trimming low-quality (Q-Score <20) nucleotides and potential human contaminants were removed using Bowtie2 (version 2.2.4). Metagenomics taxonomical annotation was performed using DIAMOND (V0.9.9.110) and used a library of NCBI blast to provide pan-microbial (bacterial, archaeal, viral and eukaryotic) quantification at the species level. All samples were compared and analyzed at the species level by relative abundance. The shotgun metagenomic DNA sequencing data from fecal samples were obtained from Beijing Novogene Biotechnology Co., Ltd, China.

### ELISA

The intestinal inflammatory markers (α1-AT, CALP and LF) and the pro-inflammatory cytokines (TNF-α, IL-6) level in mice were detected using mouse ELISA kits (E-EL-M2428c, E-EL-M1143c and E-EL-M0746c, E20230613–20852B, E20230613–20188B, (Elabscience Biotechnology Co., Ltd, China and Shanghai Enzyme-linked Biotechnology Co., Ltd. China)) according to the manufacturer’s instructions. First, Reference Standard & Sample Diluent (100 μL) was added to the blank well, and Reference Standard or test sample (100 μL) was added to the rest well, respectively. Then the plate was coated with Plate Sealer for 90 min at 37°C. Biotinylated Detection Ab Diluent (100 μL, prepared within 20 min before use) was applied to each well after discarding the fluid, and the plate was coated with plate sealer for 60 min at 37°C. After liquid drainage, the plate was rinsed 3 times and soaked for 30 s each time (approximately 350 μL per well). Finally, the plate was dried by shaking and patting on absorbent paper. The prepared HRP Conjugate Diluent (100 μL, prepared within 20 min before use in the dark) was added to each well, and the plate was coated with plate sealer for 30 min at 37°C. Substrate reagent (TMB, 90 μL) was applied to each well, and the plate was coated with Plate Sealer for 15 min at 37°C. Subsequently, 50 μL stop solution was added and the absorbance of all wells was measured at 450 nm using a Multiskan Microplate Reader (Thermo Fisher Scientific, USA). The unused reagents were stored in the refrigerator at the specified temperature when the experiment was finished.

### Histopathology analysis

HE staining was performed to evaluate morphology and inflammatory infiltration of the gastrointestinal tract from mice, using the intestinal damaging score based on the criteria of Chiu’s method.^68^ For immunohistochemistry, the 4-μm tissue sections were deparaffinized and boiled in sodium citrate buffer (10 mM sodium citrate, 0.05% Tween-20, pH 6.0) at 95–100°C for 20 min to perform antigen retrieval. Following peroxidase blocking, sections were washed multiple times in PBS and incubated in serum blocking solution for 1 h (0.5% BSA and 3% goat serum). Then the sections were inoculated with primary antibody overnight at 4°C. After being rinsed in PBS, the sections were inoculated with horseradish peroxidase-conjugated secondary antibody for 2 h at room temperature. Then, the sections were again washed 3 times for 5 min and stained with 3,3’-diaminobenzidine.

For immunofluorescence staining, the paraffin sections were treated with sodium citrate buffer to perform antigen retrieval after deparaffinization and rehydration. The sections were cooled for 30 min at room temperature and then incubated with 5% BSA for 1 h. Afterward, the sections were inoculated with appropriate dilutions of primary antibodies in PBS overnight at 4°C. Then the tissue sections were rinsed five times for 5 min at room temperature, and incubated with fluorescence-conjugated secondary antibodies for 3 h at room temperature. The tissue sections were washed three times with PBS at room temperature and then incubated with 1:1000 Hoechst 33,258 for 7 min. Tissue sections were again rinsed 3 times for 5 min at room temperature and mounted with glycerol and glass coverslips. Preparations were stored at −20°C until images were acquired using a B×43Upright Microscope (Olympus, Japan) with a DP74 camera (Olympus, Japan) or a Zeiss LSM 980 (Carl Zeiss, Germany).

### Western blot

The colon tissues of *Lrrk2*^RP/+^ mice were obtained at 15 wpfg, and the midbrain tissues of *Lrrk2*^RP/+^ mice at 24 wpfg and *Lrrk2*^RP/RP^ mice at 21 wpfg were obtained for protein extraction. The tissues were homogenized in TBS+ (50 mM Tris-HCl, pH 7.4, 175 mM NaCl and 5 mM EDTA), and a protease inhibitor mixture (Thermo Fisher Scientific, USA). The homogenized fractions were ultracentrifuged at 120,000 g at 4°C for 30 min. The supernatants were collected and the insoluble fractions were rinsed with TBS+ containing 1% Triton X-100, TBS+ containing 1 M sucrose, and RIPA buffer (Beyotime, China). After centrifugation at 120,000 g at 4°C for 20 min, the final insoluble fractions were homogenized in 8 M urea/5% SDS. Ten micrograms of insoluble and soluble fractions were separated by 12% or 15% polyacrylamide gel electrophoresis and blotted on polyvinylidene fluoride membranes (Millipore, Germany). All membranes were incubated with 5% dry skim milk in TBST (TBS with 0.1% Tween-20) for 1 h at room temperature and then the membranes were incubated with primary antibody diluted in TBST overnight at room temperature. After the membranes were again rinsed with TBST, horseradish peroxidase-conjugated secondary antibodies were used to incubate the membrane for 1 hour at room temperature. Finally, the membrane was detected with enhanced chemiluminescence (Thermo Fisher Scientific, USA). The expression levels of proteins were normalized to those of β-actin or GAPDH. Densitometry was analyzed by ImageJ software.

### HPLC analysis

HPLC analysis was used to evaluate the dopamine and DOPAC levels.^69^ The midbrain, colon and feces from mice were weighed and mixed with 1 mL methanol. After centrifugation at 12,000 rpm for 10 min, 50 μL of supernatant and 150 μL methanol were mixed and then analyzed by HPLC. The standard curve solution was used to determine a series of concentration gradients. Chromatograms and integration of dopamine were recorded by Xcalibur 3.0 software (Thermo Fisher Scientific, USA) for linear regression with a weighted coefficient of 1/X2.

### Terminal deoxynucleotidyl transferase (TdT)-mediated dUTP nick labeling (TUNEL) staining

For terminal deoxynucleotidyl transferase dUTP nick-end labeling (TUNEL) assays, DNA damage analysis was performed using a Colorimetric TUNEL Apoptosis Assay Kit (Cat#: C1098; Beyotime). Briefly, the sections were rehydrated and dewaxed before the TUNEL staining. Then the sections were digested with proteinase K (37°C) for 30 min. Following PBS washing, the sections were blocked with 3% H_2_O_2_ for 25 min at room temperature. Samples were incubated with the TUNEL reaction mixture at 37°C for 60 minutes and were stained with streptavidin-HRP at room temperature for 30 minutes. Afterward, the samples were observed under a fluorescence microscope to observe the expression of TUNEL-positive cells in the SNc. TUNEL-positive cells were brown.^70,71^

### Statistical analysis

All statistical analyses of the data were performed using SPSS 21.0 (IBM, USA) and GraphPad Prism software 8.0 software (GraphPad Software, USA). Data from HE, immunohistochemistry and immunofluorescence were analyzed by ImageJ software (US National Institutes of Health). The PERMANOVA test was performed for Bray-Curtis dissimilarity metrics using the adonis function in the R package. Microbial relative abundance was normalized by log transformation and the different features in each group were revealed by density ridgeline plots. The results of metagenomic data analysis were visualized using ggplot2 in R studio (version 4.0.2). The gut microbiome difference of participants was investigated using covariate-adjusted random coefficient regressions. Student’s t test was used for comparison between two groups, while one-way ANOVA either Tukey’s or Sidak’s multiple comparisons was used for multiple groups, when these data were distributed normally. Normal tests and homogeneity tests of variance for all continuous variables were made before the analysis. Differences between mouse groups were established using an unpaired two-sided t-test, two-way ANOVA or one-way ANOVA with Tukey’s multiple comparison test. Survival comparisons were made by the log-rank (Mantel-Cox) test whose *P* value was significant. The Benjamini-Hochberg correction was performed for multiple comparison corrections. *P* < .05 was considered significantly different from statistical analysis.

## Supplementary Material

Supplemental Material

## Data Availability

Metagenomic data were analyzed using custom software that is available at CRAN (https://cran.r-project.org/web/packages/pheatmap/index.html, http://CRAN.R-project.org/package=ggplot2). All relevant data supporting the findings of this study are either included within the article and its Additional Information files or are available upon request from the corresponding author. The mouse model will be made available on request.
