# Supplementary material for: Escherichia coli triggers α-synuclein pathology in the LRRK2 transgenic mouse model of PD
Source: Gut Microbes. 2023 Nov 27;15(2):2276296. doi: 10.1080/19490976.2023.2276296 (PMC10730176; doi:10.1080/19490976.2023.2276296)
Supplement: Supplemental Material [file KGMI_A_2276296_SM7151.docx]

***Escherichia* *coli*** **triggers α-synuclein pathology in the *LRRK2* transgenic mouse model of PD**

**Supplemental material**

**Supplementary Figure S1****.** PD-like phenotypes are triggered by *E*. *coli* and reversed by FMT in *Lrrk2*^RP/RP^ mice. (a) Generation of *Lrrk2* R1628P knock-in mouse model. (b) Timeline schematic of experimental design showing the procedures of *E*. *coli* administration and FMT in *Lrrk2*^RP/RP^. (c) Representative images of HE staining in the colon of *Lrrk2*^RP/RP^ PBS, *Lrrk2*^RP/RP^ *E*. *coli*, *Lrrk2*^RP/RP^ *E*. *coli*-PBS and *Lrrk2*^RP/RP^ *E*. *coli*-FMT (left). Quantification of the histological scores at 12, 15 and 21wpfg, respectively (right). (d) Representative images of colon length in *Lrrk2*^RP/RP^ PBS, *Lrrk2*^RP/RP^ *E*. *coli*, *Lrrk2*^RP/RP^ *E*. *coli*-PBS and *Lrrk2*^RP/RP^ *E*. *coli*-FMT at 21 wpfg (left), and quantification (right). (e) Representative immunofluorescence images of E-cadherin (green) and curli (magenta) in the colon of *Lrrk2*^RP/RP^ PBS, *Lrrk2*^RP/RP^ *E*. *coli*, *Lrrk2*^RP/RP^ *E*. *coli*-PBS and *Lrrk2*^RP/RP^ *E*. *coli*-FMT (left). Quantification of E-cadherin (up) and curli (down) fluorescence intensity at 12, 15 and 21 wpfg, respectively. (f) Representative immunohistochemical images of pα-syn in the colon, 10N, SNc, STR and PFC of *Lrrk2*^RP/RP^ PBS, *Lrrk2*^RP/RP^ *E*. *coli*, *Lrrk2*^RP/RP^ *E*. *coli*-PBS and *Lrrk2*^RP/RP^ *E*. *coli*-FMT (left). Quantification of pα-syn immunoreactivity at 12, 15 and 21 wpfg, respectively (right). (g) Representative immunohistochemical images of TH-positive neurons in SNc in *Lrrk2*^RP/RP^ PBS, *Lrrk2*^RP/RP^ *E*. *coli*, *Lrrk2*^RP/RP^ *E*. *coli*-PBS and *Lrrk2*^RP/RP^ *E*. *coli*-FMT (left). Quantification of TH immunoreactivity in SNc from mice at 21 wpfg, respectively (right). (h) HPLC analysis of dopamine levels in the midbrain, colon and feces of *Lrrk2*^RP/RP^ PBS, *Lrrk2*^RP/RP^ *E*. *coli*, *Lrrk2*^RP/RP^ *E*. *coli*-PBS and *Lrrk2*^RP/RP^ *E*. *coli*-FMT at 21 wpfg. (i) Kaplan-Meier survival curves of *Lrrk2*^RP/RP^ PBS, *Lrrk2*^RP/RP^ *E*. *coli*, *Lrrk2*^RP/RP^ *E*. *coli*-PBS and *Lrrk2*^RP/RP^ *E*. *coli*-FMT. (j) Behavioral analysis of *Lrrk2*^RP/RP^ PBS, *Lrrk2*^RP/RP^ *E*. *coli*, *Lrrk2*^RP/RP^ *E*. *coli*-PBS and *Lrrk2*^RP/RP^ *E*. *coli*-FMT at 9, 12, 14, 15, 18, and 21 wpfg, respectively. Experimental data for (c-j) were obtained from 7 independent mice, with similar results obtained. Data are shown as the mean ± SD with *P* values by one-way (d, g, h) or two-way (c, e, f, j) ANOVA with Tukey’s multiple comparison’s test and Log-rank (Mantel-Cox) test (i) **P* < 0.05, ***P* < 0.01, ****P* < 0.001, *****P* < 0.0001(*Lrrk2*^RP/RP^ PBS versus *Lrrk2*^RP/RP^ *E*. *coli*) (up); ^#^*P* < 0.05, ^##^*P* < 0.01, ^###^*P* < 0.001, ^####^*P* < 0.0001 (*Lrrk2*^RP/RP^ *E*. *coli*-PBS versus *Lrrk2*^RP/RP^ *E*. *coli*-FMT) (down). Scale bar, 20 μm (c, e, f), 50 μm (g).

**Supplementary Figure S2****.** Heatmap of pathological α-syn burden. Semi-quantitative analysis of the burden of α-syn pathology in the intestine and brain of *Lrrk2*^RP/+^ *E*. *coli* at 9, 11, 13, 15 and 24 wpfg (a) and *Lrrk2*^RP/RP^ *E*. *coli* at 6, 8, 10, 12 and 21 wpfg (b). Percentage area occupied by pathological α-syn is shown as the heatmap. The names of the associated areas are plotted onto identical maps at the bottom.

**
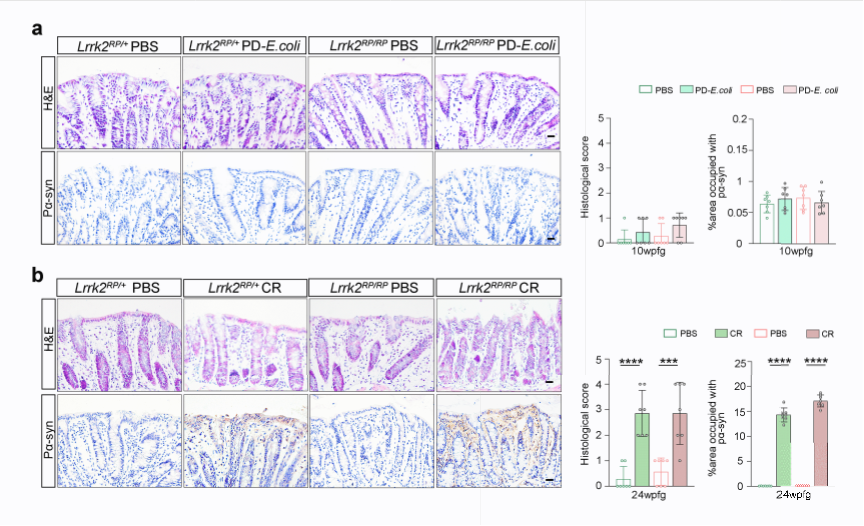
**

**Supplementary Figure S3**. (a) Representative images of HE staining in the colon of *Lrrk2*^RP/+^ PBS, *Lrrk2*^RP/+^ PD-*E. coli*, *Lrrk2*^RP/RP^ PBS, and *Lrrk2*^RP/RP^ PD-*E. coli*, respectively (up, left). Quantification of the histological scores at 10 wpfg, respectively (up, right). (b) Representative immunohistochemical images of pα-syn in the colon of *Lrrk2*^RP/+^ PBS, *Lrrk2*^RP/+^ CR, *Lrrk2*^RP/RP^ PBS, and *Lrrk2*^RP/RP^ CR, respectively (down, left). Quantification of pα-syn used the % area occupied with pα-syn in the colon at 24wpfg (down, right). Experimental data for (a-b) were obtained from 7 independent mice, with similar results obtained. **p* < 0.05, ***p* < 0.01, ****p* < 0.001, *****p* < 0.0001. Scale bar, 20 μm (a,b).

**Supplementary Figure S4****.** Mut curli *E*. *coli*, *K*. *pneumoniae*, *P*. *mirabilis*, or DSS administration to *Lrrk2*^RP/+^ mice and *E*. *coli* administration to WT and *Snca* A53T transgenic mice fails to trigger pα-syn pathology. (a) Timeline schematic of experimental design showing the procedures of oral gavage with mut-curli *E*. *coli*, *K*. *pneumoniae*, *P*. *mirabilis* or DSS to *Lrrk2*^RP/+^ mice, and with *E*. *coli* to WT and *Snca* A53T mice, respectively. (b) Representative images of HE staining in the colon of WT *E*. *coli*, *Lrrk2*^RP/+^*E*. *coli,* and *Lrrk2*^RP/RP^ *E*. *coli*, respectively (up, left). Quantification of the histological scores at 24 wpfg, respectively (up, right). Representative immunohistochemical images of pα-syn in the colon of WT *E*. *coli*, *Lrrk2*^RP/+^*E*. *coli,* and *Lrrk2*^RP/RP^ *E*. *coli*, respectively (down, left). Quantification of pα-syn used the % area occupied with pα-syn in the colon at 24wpfg (down, right). (c) Representative immunofluorescence images of E-cadherin (green) and curli (magenta) in the colon of *Lrrk2*^RP/+^ mice treated with mut curli *E*. *coli*, *K*. *pneumoniae*, *P*. *mirabilis* or DSS at 24 wpfg, and *Snca* A53T mice treated with *E*. *coli* at 24 wpfg (left). Quantification of E-cadherin (up) and curli (down) fluorescence intensity, respectively (right). (d) Representative images of HE staining in the colon of *Lrrk2*^RP/+^ mice treated with mut curli *E*. *coli*, *K*. *pneumoniae*, *P*. *mirabilis* or DSS at 24 wpfg, and *Snca* A53T mice treated with *E*. *coli* at 24 wpfg (left). Quantification of histological score (right). (e) Representative immunohistochemical images of pα-syn in the colon of *Lrrk2*^RP/+^ mice treated with mut curli *E*. *coli*, *K*. *pneumoniae*, *P*. *mirabilis* or DSS, and *Snca* A53T mice treated with *E*. *coli* (left). Quantification of pα-syn used the % area occupied with pα-syn in the colon at 24 wpfg (right). Experimental data for (b-e) were obtained from 7 independent mice, with similar results obtained. Data are shown as the mean ± SD with *P* values by one-way ANOVA with Tukey’s multiple comparison’s test (b-e). **p* < 0.05, ***p* < 0.01, ****p* < 0.001, *****p* < 0.0001. Scale bar 20 μm (b-d).

**Supplementary Figure S5****.** No obvious changes of these α-syn phosphorylation related protein kinases were detected in *Lrrk2*^RP/+^ *E*. *coli*-EVs mice. Representative immunoblot images of PLK2/3, PKR, PKR phosphorylation, and GRK3/5/6 levels in the soluble fractions of colon in *Lrrk2*^RP/+^ *E*. *coli*-EVs, *Lrrk2*^RP/+^ mut *E*. *coli*- EVs, *Lrrk2*^RP/+^ PBS-EVs and WT *E*. *coli*-EVs, WT mut *E*. *coli*-EVs, WT PBS-EVs at 8 weeks after injection (left) and quantification (right). Experimental data of this study was obtained from 7 independent mice, with similar results obtained. Data are shown as the mean ± SD with *P* values by one-way ANOVA with Tukey’s multiple comparison’s test. **p* < 0.05, ***p* < 0.01, ****p* < 0.001, *****p* < 0.0001.

**Supplementary Figure S6****.** A proposed schematic representation of the interaction of *LRRK2* variants induce intestinal impairment and *E*. *coli* produced curli contributing to *LRRK2*-associated sPD. Intestinal mucosal barrier disruption in *LRRK2* risk variants carriers increase contents leakage into intestinal wall from intestinal lumen, e.g., *E*. *coli* and curli. Dysbiosis and increased *E*. *coli* induce a great deal of curli, which may trigger the deposition of pathological α-syn. Pathological α-syn aggregates are capable of prion-like propagation to the brain via the gut-brain axis to cause neurodegeneration in *LRRK2*-associated sPD.

**Supplementary Table S1. The top 20 fecal microbial species contrasting in samples from *LRRK2*+/sPD+ compared with *LRRK2*+/sPD- and HCs.**

| **Genus** | **HCs (n =30)** | ***LRRK2+/sPD-* (n =21)** | ***LRRK2+/sPD+* (n=51)** | **Adjust *P*^a^ value** | **Adjust *P*^b^ value** | **Adjust *P*^c^ value** |
| --- | --- | --- | --- | --- | --- | --- |
| ***Escherichia coli*** | 0.0007(0.0003 - 0.0073) | 0.0004(0.0002 - 0.0054) | 0.0291(0.0079 - 0.053) | 0.9955 | <.0001 | <.0001 |
| ***Faecalibacterium prausnitzii*** | 0.0238(0.0103 - 0.039) | 0.0276(0.0155 - 0.0382) | 0.0046(0.0008 - 0.0175) | 0.9874 | <.0001 | 0.0003 |
| ***Prevotella copri*** | 0.001(0.0001 - 0.0156) | 0.0028(0.0002 - 0.0362) | 0.0003(0 - 0.0051) | 0.9961 | 0.159 | 0.2713 |
| ***Chlamydia trachomatis*** | 0.0004(0.0002 - 0.0051) | 0.0006(0.0003 - 0.0059) | 0.0006(0.0001 - 0.003) | 0.3676 | 0.9994 | 0.2892 |
| ***Lactobacillus salivarius*** | 0(0 - 0.0001) | 0(0 - 0.0001) | 0.0005(0 - 0.0068) | 0.9919 | 0.115 | 0.2293 |
| ***Bifidobacterium adolescentis*** | 0.0007(0.0003 - 0.0047) | 0.001(0.0003 - 0.0026) | 0.0026(0.0003 - 0.0083) | 0.6534 | 0.3388 | 0.9602 |
| ***uncultured crAssphage*** | 0(0 - 0.0001) | 0.0001(0 - 0.0044) | 0(0 - 0.0012) | 0.6368 | 0.6175 | 0.9842 |
| ***Lactobacillus gasseri*** | 0(0 - 0) | 0(0 - 0) | 0(0 - 0.0017) | 1 | 0.2868 | 0.3721 |
| ***Bifidobacterium pseudocatenulatum*** | 0.0019(0.0001 - 0.0062) | 0.0003(0.0001 - 0.0024) | 0.0025(0.0009 - 0.0074) | 0.4674 | 0.8982 | 0.215 |
| ***Bifidobacterium longum*** | 0.001(0.0004 - 0.0045) | 0.0008(0.0003 - 0.0023) | 0.0038(0.0009 - 0.009) | >0.9999 | 0.0525 | 0.1673 |
| ***Eubacterium* sp. CAG:180** | 0.0001(0 - 0.0073) | 0.0001(0 - 0.0031) | 0(0 - 0.0049) | 0.9995 | 0.5918 | 0.686 |
| ***Lactobacillus mucosae*** | 0(0 - 0) | 0(0 - 0.0001) | 0.0004(0 - 0.0032) | 0.9934 | 0.8035 | 0.8965 |
| ***[Eubacterium] rectale*** | 0.0013(0.0007 - 0.0098) | 0.002(0.0007 - 0.0108) | 0.0002(0.0001 - 0.0028) | 0.459 | 0.3077 | 0.0276 |
| ***Bacteroides vulgatus*** | 0.0012(0.0006 - 0.0042) | 0.0023(0.0008 - 0.0047) | 0.0012(0.0004 - 0.0047) | 0.859 | 0.4687 | 0.8896 |
| ***uncultured Clostridium* sp.** | 0.0053(0.0029 - 0.0072) | 0.0047(0.0033 - 0.0065) | 0.0017(0.0004 - 0.0039) | 0.9878 | 0.0047 | 0.0075 |
| ***Dorea longicatena*** | 0.0031(0.0014 - 0.0082) | 0.0034(0.0022 - 0.0069) | 0.001(0.0002 - 0.0033) | 0.6319 | 0.0011 | 0.0664 |
| ***Klebsiella pneumoniae*** | 0.0002(0 - 0.0009) | 0.0001(0 - 0.0013) | 0.0011(0.0003 - 0.0021) | 0.9863 | 0.6658 | 0.6169 |
| ***Romboutsia timonensis*** | 0.001(0.0003 - 0.0033) | 0.0013(0.0003 - 0.0041) | 0.0002(0 - 0.0011) | 0.7075 | 0.4135 | 0.1191 |
| ***Holdemanella biformis*** | 0.0002(0.0001 - 0.0024) | 0.0002(0.0001 - 0.0029) | 0.0001(0 - 0.001) | 0.7132 | 0.9258 | 0.851 |
| ***Collinsella aerofaciens*** | 0.0035(0.0011 - 0.006) | 0.0025(0.0013 - 0.0059) | 0.0011(0.0001 - 0.003) | 0.9084 | 0.0299 | 0.1605 |

Data presented as median (interquartile range); *LRRK2* = Leucine-rich repeat kinase 2; sPD = sporadic Parkinson’s disease; HCs = healthy controls; *P*^a^: inter-group comparisons (HCs vs. *LRRK2*+/sPD-); *P*^b^: inter-group comparisons (HCs vs. *LRRK2*+/sPD+); *P*^c^: inter-group comparisons (*LRRK2*+/sPD- vs. *LRRK2*+/sPD+).
